# Five-lncRNA signature in plasma exosomes serves as diagnostic biomarker for esophageal squamous cell carcinoma

**DOI:** 10.18632/aging.103559

**Published:** 2020-06-28

**Authors:** Zichen Jiao, Ao Yu, Weiwei Rong, Xiaofeng He, Ke Zen, Minke Shi, Tao Wang

**Affiliations:** 1 Department of Cardiothoracic Surgery, Nanjing Drum Tower Hospital, Nanjing Medical University, Nanjing, China; 2 School of Life Science, Nanjing University, Nanjing, China; 3 Department of Cardiothoracic Surgery, Nanjing Drum Tower Hospital, Medical School of Nanjing University, Nanjing, China

**Keywords:** ESCC, plasma, exosome, lncRNA, biomarker

## Abstract

Changes in expression of long non-coding RNAs (lncRNAs) in plasma exosomes can be useful for diagnosis of cancer patients. Here, we conducted a four-stage study to identify plasma exosome lncRNAs with diagnostic potential in esophageal squamous cell carcinoma (ESCC). First, plasma exosome lncRNA expression profiles were examined in ESCC patients, esophagitis patients, and healthy controls using RNA sequencing. Differentially expressed plasma exosome lncRNAs from the lncRNA expression profile were then evaluated by qRT-PCR in a large cohort of ESCC patients, esophagitis patients, and healthy controls. Expression levels of the lncRNAs NR_039819, NR_036133, NR_003353, ENST00000442416.1, and ENST00000416100.1 were significantly higher in exosomes from ESCC patients than non-cancer controls. We also confirmed that levels of these five plasma exosome lncRNAs decreased markedly in ESCC patients after surgery. Our results suggest that these five exosome lncRNAs may serve as highly effective, noninvasive biomarkers for ESCC diagnosis.

## INTRODUCTION

Esophageal squamous cell carcinoma (ESCC) is one of the most common digestive tract tumors worldwide and is associated with high morbidity and mortality, especially in Asia [1]. Despite improvements in surgical techniques and perioperative management, the 5-year survival rate of ESCC is less than 30% [1]. Currently, the most frequently examined biomarkers for ESCC diagnosis are serum squamous cell carcinoma antigen (SCCA), carbohydrate antigen (CA) 19–9, and carcinoembryonic antigen (CEA). However, these biomarkers are relatively insensitive and inaccurate [1]. Novel effective biomarkers for ESCC are therefore urgently needed.

Long non-coding RNAs (lncRNAs) are longer than 200 bases in length and do not code for proteins. An increasing number of studies indicate that lncRNAs can affect cancer initiation, progression, and metastasis by modulating oncogenic and tumor-suppressing pathways [2]. Recently, several lncRNAs have been identified that are secreted via exosomes from tumor tissues into the plasma, and dysregulated expression of these lncRNAs in exosomes could potentially serve as a diagnostic or prognostic biomarker for many tumors, such as liver and lung cancer [3, 4]. However, few studies have comprehensively explored exosome lncRNA profiles in plasma from ESCC patients.

Here, we conducted a four-stage study to identify plasma exosome lncRNAs with diagnostic potential in ESCC. First, lncRNA expression profiles were characterized in plasma exosomes from ESCC patients using RNA-sequencing. Five lncRNAs, NR_039819, NR_036133, NR_003353, ENST00000442416.1, and ENST00000416100.1, were found to be significantly increased in the ESCC patients. Expression of these five lncRNAs was then evaluated by qRT-PCR in a large cohort of ESCC patients, esophagitis patients, and healthy controls. Levels of all five lncRNAs were significantly increased in exosomes from ESCC patients and decreased in those patients after surgery. Our results therefore indicate that these five exosome lncRNAs might serve as useful biomarkers for ESCC diagnosis.

## RESULTS

### Characterization of plasma exosomes

**Figure 1:**
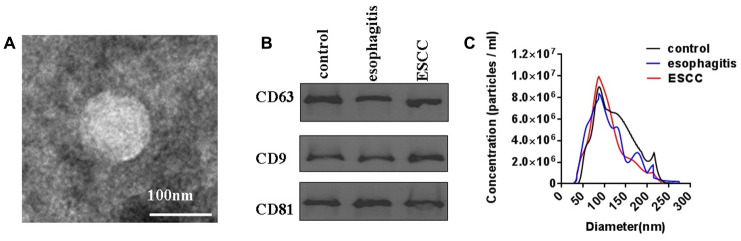
**Characterization of plasma exosomes from ESCC patients, esophagitis patients, and healthy controls.** (**A**) Shape and structure of plasma exosomes isolated by ultracentrifugation under TEM. (**B**) Western blots of exosome membrane markers CD63, CD9, and CD81. (**C**) Sizes of plasma exosomes from ESCC patients, esophagitis patients, and healthy controls were analyzed by NTA.

Because exosome purity affects the variability of exosome lncRNA analysis results, TEM, Western blotting, and nanoparticle tracking analysis were used to characterize exosomes isolated from the plasma of ESCC patients, esophagitis patients, and healthy controls. As shown in Figure 1A, exosomes were confirmed to have sizes falling within the typical range as well as normal rounded membrane-bound morphologies using TEM. Expression of the established exosome markers CD63, CD9, and CD81 was then characterized through Western immunoblotting analysis (Figure 1B). Finally, nanoparticle tracking analysis was used to confirm that the exosome diameters were approximately 100 nm as previously reported (Figure 1C) [5]. These results demonstrated that exosomes were successfully extracted from plasma.

### Identification of exosome lncRNA profiles using Illumina HiSeq 2500

**Figure 2:**
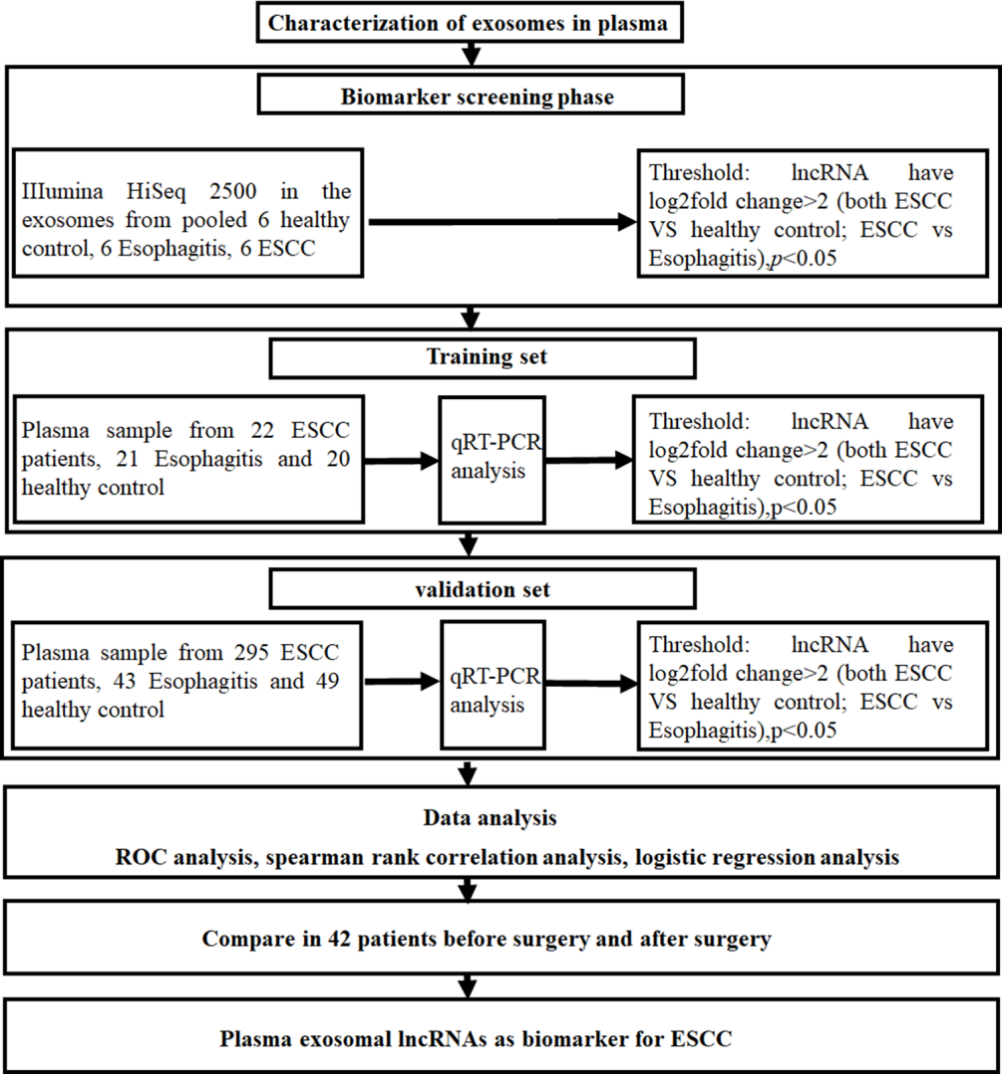
**Experimental design flowchart.**

**Figure 3:**
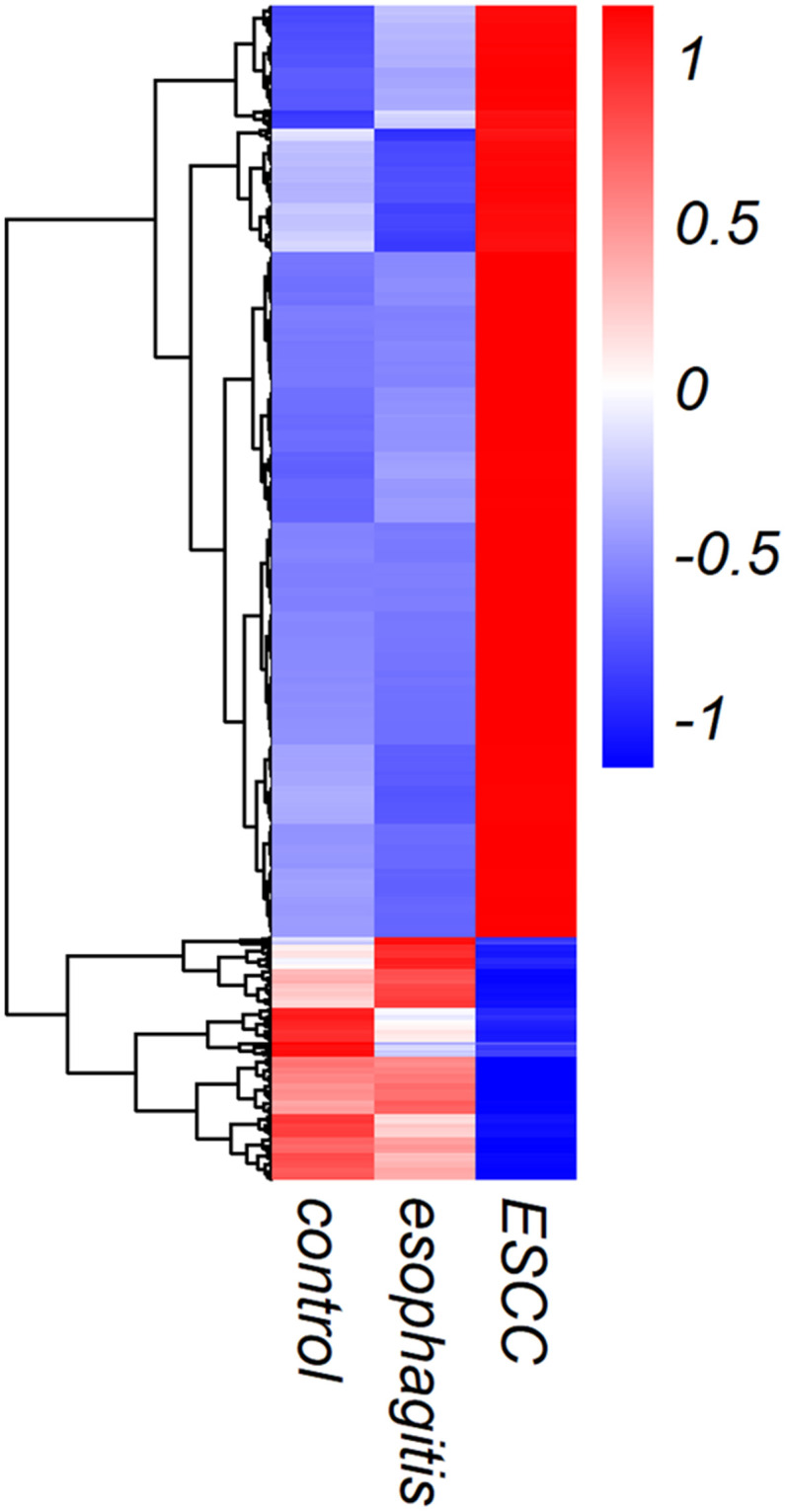
**Heat map showing lncRNA expression in exosomes from ESCC patients, esophagitis patients, and healthy controls.**

In order to identify plasma exosome lncRNAs with diagnostic potential in ESCC, a four-stage study was conducted (Figure 2). Exosome lncRNAs in pooled plasma from ESCC patients, esophagitis patients, and healthy controls were profiled using Illumina HiSeq 2500 analysis; each pooled sample included plasma from ten patients. The heatmap shown in Figure 3 revealed distinct lncRNA expression patterns for plasma exosomes from ESCC patients, esophagitis patients, and healthy controls. LncRNAs with a median number of reads > 100 and with a ≥ 2-fold change (*P* < 0.05) between ESCC patients and esophagitis patients/healthy controls were selected as potential biomarkers. The following five lncRNAs were significantly increased in ESCC patients compared to esophagitis patients and healthy controls: NR_039819, NR_036133, NR_003353, ENST00000442416.1, and ENST00000416100.1 (Table 1).

**Table 1:** LncRNAs with reads > 100, log_2_(fold change) ≥ 2, and *P* < 0.05 in Illumina HiSeq 2500 biomarker screening.

**Transcript**	**Gene**	**Control**	**Esophagitis**	**ESCC**	**log_2_(fold change)**	***p-value***	**log_2_(fold change)**	***p-value***
					**(ESCC *vs*. control)**	**(ESCC *vs*. control)**	**(ESCC *vs*. esophagitis)**	**(ESCC *vs*. esophagitis)**
NR_039819	pre-MIR4672	0.3807	0.4221	7.0044	4.2013	0.0237	4.0527	0.0176
NR_036133	pre-MIR323B	0.7824	0.2191	12.2192	3.9652	0.0019	5.8015	0.0012
NR_003353	SNORD115-38	0.8659	1.1275	10.7715	3.6369	0.0201	3.2561	0.0137
ENST00000442416.1	LINC02832	0.8827	1.1183	7.2648	3.0409	0.0000	2.6997	0.0001
ENST00000416100.1	AC093734.1	1.7574	1.5737	7.1267	2.0198	0.0064	2.1791	0.0034

### qRT-PCR confirmed increased plasma exosome lncRNA levels in ESCC

**Figure 4:**
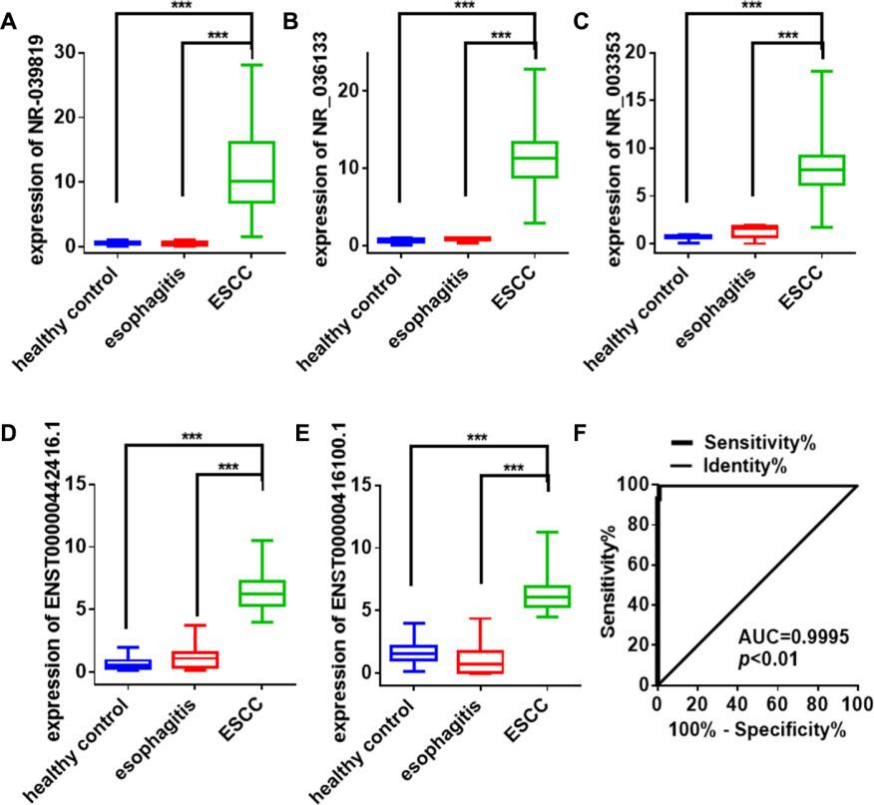
**Exosome lncRNA expression signature for ESCC diagnosis.** (**A**–**E**) Differences in plasma exosome lncRNAs levels in ESCC patients, esophagitis patients, and healthy controls. Each point represents the mean of triplicate samples. Each *P*-value was calculated with a nonparametric Mann-Whitney test. ***P<0.001. (**F**) ROC curve analysis of the combined lncRNA panel in exosomes from ESCC patients and non-cancer controls (including healthy controls and esophagitis patients). Each value is the mean±SD; ***P <0.001.

qRT-PCR using the Illumina HiSeq 2500 was performed to confirm increased expression the five potential lncRNA biomarker candidates identified above in plasma exosomes of ESCC patients (n=22) compared to esophagitis patients (n=21) and healthy volunteers (n=20) in the training set. As expected, plasma exosome NR_039819, NR_036133, NR_003353, ENST00000442416.1, and ENST00000416100.1 expression was at least 2-fold higher in ESCC patients than in esophagitis patients and healthy volunteers (*P* < 0.05). Next, we evaluated these five lncRNAs in a larger cohort consisting of 295 ESCC patients, 43 esophagitis patients, and 49 healthy volunteers. Consistent with previous results, NR_039819, NR_036133, NR_003353, ENST00000442416.1, and ENST00000416100.1 expression was higher in plasma exosomes from ESCC patients than in the other groups (*P* < 0.001, Figure 4A–4E). A logistic regression model analysis of the combined diagnostic value of these five lncRNAs revealed an AUC of 0.9995 (*P* < 0.001) (Figure 4F). Taken together, these results suggest that these five lncRNAs could serve as useful and stable diagnostic biomarkers for ESCC.

### Comparison of plasma exosome lncRNAs before and after surgery

**Figure 5:**
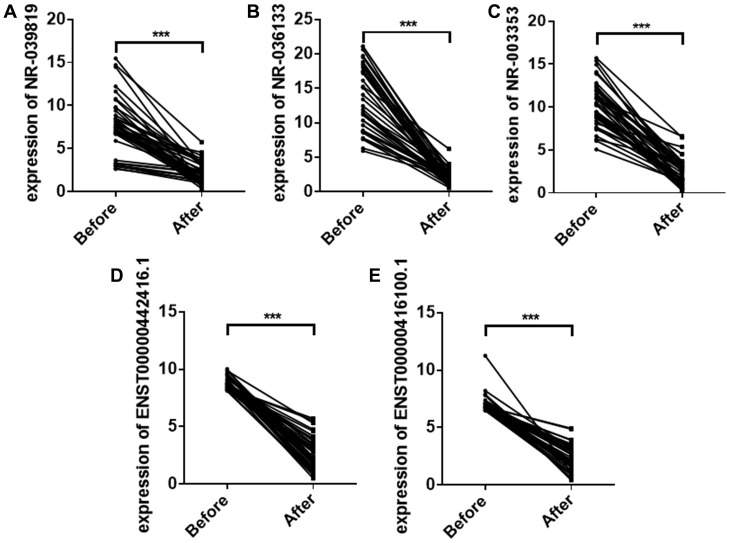
Plasma exosome NR_039819 (**A**) NR_036133 (**B**) NR_ 003353 (**C**) ENST00000442416.1(**D**) and ENST0000041 6100.1 (**E**) levels in ESCC patients before surgery and after surgery. Each point represents the mean of triplicate samples. Each P-value was calculated with a nonparametric Mann-Whitney test. ***P<0.001.

To investigate whether the five plasma exosome lncRNAs identified above could be used to monitor treatment efficacy in ESCC patients, we collected plasma samples from 42 stage I ESCC patients before and one month after surgery. Plasma exosome NR_039819, NR_036133, NR_003353, ENST00000442416.1, and ENST00000416100.1 levels decreased significantly one month after surgery compared to levels before surgery (Figure 5).

## DISCUSSION

Cell-free nucleic acids are detectable in plasma or serum exosomes from tumor patients and might therefore serve as novel biomarkers for tumor diagnosis [7]. Although numerous studies have focused on miRNAs in these exosomes as potential biomarkers for ESCC diagnosis and prognosis prediction, several recent studies have examined plasma exosome lncRNA levels in ESCC [8–11].

In this study, we screened plasma exosome lncRNAs in ESCC patients using the Illumina HiSeq 2500. LncRNAs with a mean number of reads greater than100 and a greater than 2-fold change in ESCC patients compared to esophagitis patients and healthy volunteers were further evaluated in two large independent patient cohorts by qRT-PCR. The results demonstrated that plasma exosome NR_039819, NR_036133, NR_003353, ENST00000442416.1, and ENST00000416100.1 levels were significantly higher in ESCC patients compared to esophagitis patients and healthy volunteers. Moreover, we also examined the expression of these five lncRNAs in exosomes from ESCC patient plasma before and after surgery. Consistent with our previous results, plasma exosome NR_039819, NR_036133, NR_003353, ENST00000442416.1, and ENST00000416100.1 expression decreased significantly after surgery. Together, these results provide strong evidence that plasma exosome lncRNAs could be used as novel diagnostic markers for ESCC.

**Figure 6:**
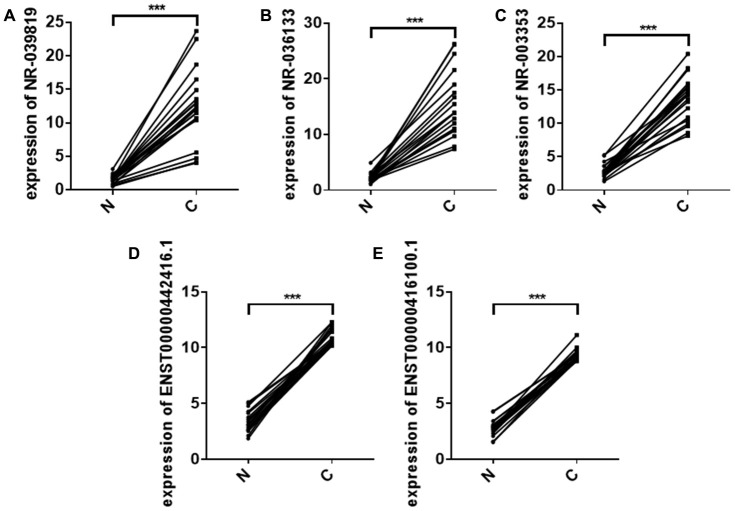
Relative NR_039819 (**A**) NR_036133 (**B**) NR_ 003353 (**C**) ENST00000442416.1 (**D**) and ENST0000041 6100.1 (**E**) levels in 20 paired non-cancerous (**N**) and ESCC cancer (**C**) tissues determined using qRT- PCR. ***P < 0.001.

LncRNAs, a relatively recently discovered type of long non-coding RNA, play critical roles in tumor development, differentiation, and formation [12]. Exosomes can mediate cellular communication in cancer by transmitting active molecules, including lncRNAs [12]. Two of the five lncRNAs identified in our study, NR_039819 and NR_036133, are precursors of MIR4672 and MIR323B. Several studies have demonstrated that exosomes can deliver miRNA precursors to target cells, allowing those precursors to reprogram the target cell transcriptome [13]. Additionally, we found that expression of these five lncRNAs was significantly increased in ESCC tumor tissues compared to distal normal tissues (Figure 6). Exosomes are known to mediate cellular communication by transmitting lncRNAs in ESCC [9, 14, 15]. For example, Li et al. confirmed that the exosomal lncRNA ZFAS1 could promote ESCC growth via the microRNA-124/STAT3 axis [9]. Exosomal FMR1-AS1 helped maintain dynamic equilibrium in cancer stem-like cells through the TLR7/NFκB/c-Myc signaling pathway [14]. Kang et al. demonstrated that exosomes facilitate transfer of the lncRNA PART1, which induces gefitinib resistance in esophageal squamous cell carcinoma via the miR-129/blc-2 axis [15]. Existing evidence therefore suggests that the five plasma exosome lncRNAs identified here might play important roles in the development and progression of ESCC. However, further studies are needed to confirm those roles.

In summary, our results suggest that increased expression of the plasma exosome lncRNAs NR_039819, NR_036133, NR_003353, ENST00000442416.1, and ENST00000416100.1 might serve as a useful tumor biomarker for ESCC diagnosis.

## MATERIALS AND METHODS

### Patient characteristics

**Figure 7:**
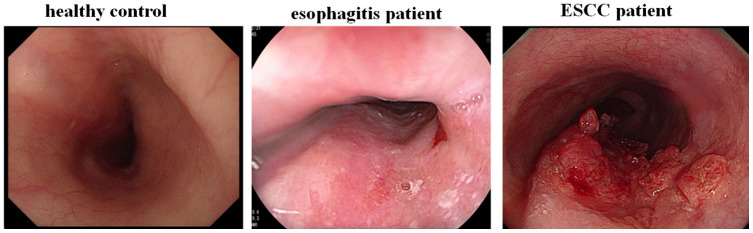
**Endoscopic examination of ESCC patients, esophagitis patients and healthy controls.**

Plasma samples from 317 untreated ESCC patients, as well as 64 esophagitis patients and 69 healthy controls who were age-, sex-, and ethnicity-matched with the ESCC patients, were included in this study. Paired preoperative and postoperative plasma samples (n = 42) were collected from stage I ESCC patients before surgery and one month after resection. Twenty pairs of ESCC tissues and adjacent non-cancerous tissues were collected from patients who were diagnosed with ESCC at the Nanjing Drum Tower Hospital. The demographic and clinical characteristics of the patients and healthy volunteers are summarized in Table 2. Esophagogastroduodenoscopies were performed for all subjects (Figure 7). A lack of abnormal mucosa, ulcers, or abnormal protuberances in the esophagus was confirmed in healthy controls. Esophagitis patients had rough mucosa covered with white moss and with visible submucosal blood vessels. ESCC patients had rough and congested surrounding mucosa with rigid tube walls, poor peristalsis, narrow lumens, and intraluminal protrusions. Diagnoses for non-cancer esophagitis patients and ESCC patients were confirmed by histopathology. This study was approved by the Ethics Committee of Nanjing Drum Tower Hospital, and written informed consent was obtained from all patients. All experiments were performed in accordance with relevant guidelines and regulations. Protocols were designed and performed according to the principles of the Helsinki Declaration.

**Table 2:** Demographic and clinical characteristics of ESCC patients, esophagitis patients, and healthy controls in the training and validation sets.

**Variable**	**Control (n=69)**	**Esophagitis (n=64)**	**ESCC (n=317)**
Age, years^a^	44.6(16.54)	45.3(18.25)	44.4(18.43)
Sex, n			
male	35	36	208
Female	34	28	109
Stage, n			
I-II			216
III-IV			101

^a^Age data are presented as the mean (SD).

### Plasma exosome isolation and RNA extraction

Exosomes were isolated from plasma using the Total Exosome Isolation Kit (from plasma) (Cat# 4484450, Invitrogen, USA) following the manufacturer’s protocol. Briefly, plasma samples were centrifuged at 1,000×g for 20 min, 3,000×g for 20 min, and 10,000×g for 20 min immediately after collection. The clarified plasma was then mixed with 0.5 volume of PBS and 0.3 volume of the Exosome Precipitation Reagent. The mixture was incubated at 4°C for 4 hours and then centrifuged at 10,000×g for 20 minutes. Subsequently, the pellet containing exosomes was collected and resuspended in 200 μL PBS for downstream analysis. The mirVana PARIS Kit (Ambion, Thermo Scientific, Shanghai, China) was used to extract total RNA from the exosomes.

### Characterization of exosomes isolated from plasma via transmission electron microscopy (TEM), nanoparticle tracking analysis, and Western blotting

Transmission electron microscopy (TEM) of exosomes isolated from plasma was performed as previously described [5]. A JEM-1011 scanning transmission electron microscope (Hitachi, Tokyo, Japan) was used to obtain the microphotographs. Nanoparticle tracking analysis was performed to identify exosomes isolated from plasma using the ZetaView PMX 110 (Particle Metrix, Meerbusch, Germany) according to the guidelines of the International Society for Extracellular Vesicles and the manufacturer’s protocol [5]. Western blotting analysis was used to characterize expression of the exosome protein markers CD63, CD81, and CD9 as previously described [5]. CD63, CD81, and CD9 were detected using anti-CD63, anti-CD81, and CD9 rabbit polyclonal antibodies (Abcam, USA). Bound proteins were visualized using ECL Western blotting substrate (Thermo Fisher Scientific, MA, USA) and analyzed with ImageJ software.

### Illumina HiSeq 2500 analysis

LncRNA expression profiles in plasma exosomes were evaluated using an Illumina HiSeq 2500 following the manufacturer’s protocol. LncRNA quantities were normalized and reported as reads per million (RPM). The EdgeR package [6] was used to analyze dysregulated lncRNAs and calculate associated *p* values. LncRNAs with a log_2_ (fold change) > 1 and *p* < 0.05 were classified as showing differential expression.

### qRT-PCR for lncRNAs

The qRT-qPCR assay for exosome lncRNA isolated from 1 mL plasma as previous described was performed following the manufacturer's protocol (Applied Biosystems, Shanghai, China). All reactions were performed in triplicate, and the data were analyzed using the comparative Cq method. The primers used are listed in Table 3

**Table 3:** Primers used in qRT-PCR.

**Name**	**Forward (5'-3')**	**Reverse (5'-3')**
NR_039819	GCTGCTTCTCGCCTCTGT	GGCTGTTTCGTGCCTCTG
NR_036133	TGGTACTCGGAGGGAGGTTG	GAGGTCGACCGTGTATTGGG
NR_003353	GGGTCAATGATGAGAACCTTACA	GGGCCTCAGCGTAATCCTAT
ENST00000442416.1	AGTAGTGCTCGTGAAGCGAT	ATCCCAAGGGTGATGTCAGG
ENST00000416100.1	GCCACAGGATGGGAGCTTAT	GTGGCCGTCTTCTCTCTGTG

### Statistical analyses

Statistical analyses were performed using SPSS 18.0 software and graphs were generated using GraphPad Prism 6.0. The Mann-Whitney U test was used to test the statistical significance of differences in plasma exosome lncRNA expression. Spearman correlation analysis was performed to identify correlations between lncRNA and clinical indicators. *P*-values < 0.05 was regarded as statistically significant.
